# Characterization of temporal coherence of hard X-ray free-electron laser pulses with single-shot interferograms

**DOI:** 10.1107/S2052252517014014

**Published:** 2017-10-13

**Authors:** Taito Osaka, Takashi Hirano, Yuki Morioka, Yasuhisa Sano, Yuichi Inubushi, Tadashi Togashi, Ichiro Inoue, Kensuke Tono, Aymeric Robert, Kazuto Yamauchi, Jerome B. Hastings, Makina Yabashi

**Affiliations:** a RIKEN SPring-8 Center, 1-1-1 Kouto, Sayo-cho, Sayo-gun, Hyogo 679-5148, Japan; b Department of Precision Science and Technology, Graduate School of Engineering, Osaka University, 2-1 Yamada-oka, Suita, Osaka 565-0871, Japan; c Japan Synchrotron Radiation Research Institute (JASRI), 1-1-1 Kouto, Sayo-cho, Sayo-gun, Hyogo 679-5198, Japan; d Linac Coherent Light Source, SLAC National Accelerator Laboratory, 2575 Sand Hill Road, MS 102, Menlo Park, CA 94025, USA

**Keywords:** X-ray interferometry, split-and-delay optical system, X-ray free-electron lasers, temporal coherence

## Abstract

Temporal coherence is one of the most fundamental characteristics of light, connecting to spectral information through the Fourier transform relationship between time and frequency. Interferometers with a variable path-length difference (PLD) between the two branches have widely been employed to characterize temporal coherence properties for broad spectral regimes. Hard X-ray interferometers reported previously, however, have strict limitations in their operational photon energies, due to the specific optical layouts utilized to satisfy the stringent requirement for extreme stability of the PLD at sub-ångström scales. The work presented here characterizes the temporal coherence of hard X-ray free-electron laser (XFEL) pulses by capturing single-shot interferograms. Since the stability requirement is drastically relieved with this approach, it was possible to build a versatile hard X-ray interferometer composed of six separate optical elements to cover a wide photon energy range from 6.5 to 11.5 keV while providing a large variable delay time of up to 47 ps at 10 keV. A high visibility of up to 0.55 was observed at a photon energy of 10 keV. The visibility measurement as a function of time delay reveals a mean coherence time of 5.9 ± 0.7 fs, which agrees with that expected from the single-shot spectral information. This is the first result of characterizing the temporal coherence of XFEL pulses in the hard X-ray regime and is an important milestone towards ultra-high energy resolutions at micro-electronvolt levels in time-domain X-ray spectroscopy, which will open up new opportunities for revealing dynamic properties in diverse systems on timescales from femto­seconds to nanoseconds, associated with fluctuations from ångström to nanometre spatial scales.

## Abstract 2

The temporal coherence of hard X-ray free-electron laser pulses was characterized by capturing single-shot interferograms with a versatile interferometer composed of six separate optical elements. The visibility measurements as a function of time delay revealed a mean coherence time of 5.9 ± 0.7 fs.

## 1. Introduction   

Optical interferometry using visible light is one of the most powerful methods for high-precision metrology owing to its high sensitivity to the phase of the light (Hariharan, 2007 ▸). Interferometry with hard X-rays can drastically enhance the sensitivity by three orders of magnitude due to the shorter wavelengths down to the ångström (10^−10^ m) scale. Furthermore, the high transmissivity of hard X-rays enables investigation of thick and opaque materials that cannot be probed with visible light. However, the implementation of an X-ray interferometer requires extreme stability at the sub-ångström level of the path length difference (PLD) between the two branches of the interferometer. In addition, optical elements composing the interferometer are strictly limited for hard X-rays due to their weak interaction with matter, which has been the origin of considerable difficulties in performing hard X-ray interferometry.

In 1965, Bonse and Hart achieved a marked advance in this field by developing a triple-Laue (LLL) crystal interferometer that consists of three blades acting as a splitter, mirror and analyser arranged in a monolithic block made of a perfect crystal of silicon (Bonse & Hart, 1965 ▸). This monolithic design significantly facilitates the stabilization of the PLD, although the development of X-ray interferometers with a variable PLD is still in great demand for expanding the range of interferometry applications. Of particular interest is the characterization of temporal coherence, which is one of the most fundamental properties of light and indicates the correlation between two wavefields separated longitudinally. Furthermore, the temporal coherence connects to the spectral information through the Fourier transform (FT) relationship between time and frequency (*i.e.* photon energy), and thus a PLD of 100 mm yields an ultra-high energy resolution of approximately 10 µeV, which is beyond the resolution achieved with state-of-the-art X-ray monochromators/spectrometers made of perfect crystals (Yabashi *et al.*, 2001 ▸; Shvyd’ko *et al.*, 2003 ▸). Appel & Bonse (1991 ▸) first demonstrated an X-ray Michelson interferometer in which the LLL interferometer was combined with weakly linked Bragg-case channel-cut crystals placed on a common rotational stage. They obtained interferograms with a high visibility, although the PLD range was limited to about 100 nm, which corresponds to an energy resolution of approximately 10 eV in the FT analysis. Fezzaa & Lee (2001 ▸), and recently Sakamoto *et al.* (2017 ▸), reported operation of a monolithic fourfold interferometer with a small slope of the diffracting surfaces in one branch. Although they achieved a larger PLD on the millimetre scale, the photon energies at which it operates are discrete and limited because of the utilization of three-beam-case Bragg diffractions. Tamasaku *et al.* (2003 ▸) proposed a scheme to achieve a 100 mm PLD by combining a double-Laue beam splitter and a back-scattering crystal completely separated from each other, in which the stability requirement is satisfied with the help of an intensity correlation technique. However, the back-scattering condition again restricts the photon energy.

In these studies, specific optical configurations, such as weakly linked crystals, multiple diffraction or back-scattering geometry, with limited degrees of freedom, were used to satisfy the stringent requirement for PLD stability. However, a drastic enhancement in flexibility in the optical design should allow for much broader applications. In this paper, we report a new approach of hard X-ray interferometry by capturing single-shot interferograms for a pulsed X-ray source, an X-ray free-electron laser (XFEL) (Emma *et al.*, 2010 ▸; Ishikawa *et al.*, 2012 ▸), which is analogous to interferometry with FELs from the EUV to soft X-ray spectral regimes (Mitzner *et al.*, 2008 ▸; Schlotter *et al.*, 2010 ▸; Singer *et al.*, 2012 ▸). Since the stability requirement has been drastically relieved with this approach, we were able to design and operate a versatile X-ray interferometer composed of six separate optical elements, operating over a continuous photon energy range from 6.5 to 11.5 keV with a maximum PLD of 66 mm. The validity of this scheme was verified through characterization of the temporal coherence of XFEL pulses at the SPring-8 Ångström Compact Free-Electron Laser (SACLA) (Ishikawa *et al.*, 2012 ▸).

## 2. Experimental   

**Figure 1:**
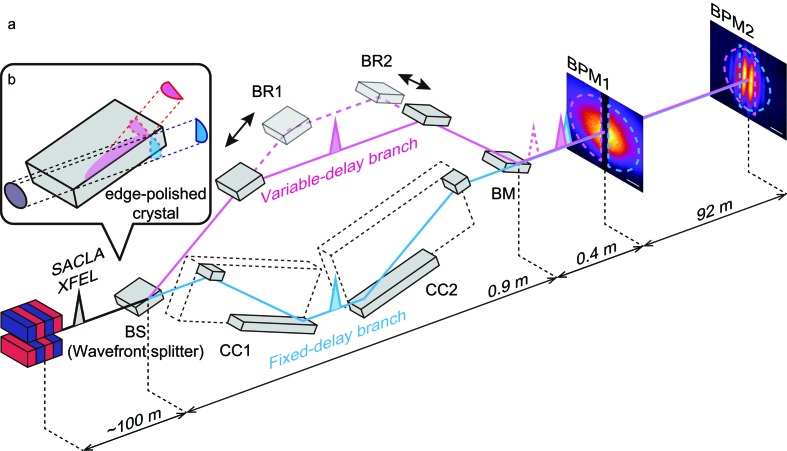
(*a*) Schematic diagram of the experimental setup with a separate six-crystal interferometer. A wavefront of a 10 keV XFEL pulse propagating through an Si(111) double-crystal monochromator (not displayed) is split into two parts by an edge-polished crystal beam splitter (BS). A conceptual sketch of the wavefront division is depicted in panel (*b*). The transmission part (blue, lower path) propagates in the fixed-delay branch through fourfold Bragg-case reflections at a set of two channel-cut crystals (CCs). The other, reflection, part (red, upper path) is reflected three more times by two movable beam reflectors (BRs) and a beam merger (BM), and recombines with the transmission part at the BM in the variable-delay branch. By introducing an angular deviation between the two beams, the two initially spatially separated split X-ray pulses are superimposed at an imaging detector (BPM2) and form interference fringes with a near-zero delay. Another imaging detector (BPM1) is used to align the optical elements in the variable-delay branch.

Fig. 1 ▸ shows a schematic of the interferometer, which consists of a variable-delay and a fixed-delay branch with six separate Bragg-case Si(220) crystals. It is often referred to as a split-and-delay optical (SDO) system (Osaka *et al.*, 2016 ▸). A similar optical system for the hard X-ray regime has been developed (Roseker *et al.*, 2009 ▸, 2011 ▸), but it operates at discrete photon energies. The wavefront of an XFEL pulse, with high transverse coherence, is split into two parts with a beam splitter (BS) made of an edge-polished crystal, as illustrated in Fig. 1 ▸(*b*). Only the part of the X-ray pulse illuminating the BS is diffracted towards the first and second Bragg beam reflectors (BR1, BR2) in the variable-delay branch, whereas the other part is transported to a pair of damage-free channel-cut crystals (CC1, CC2) (Hirano *et al.*, 2016 ▸) operating in a (+, −, −, +) geometry in the fixed-delay branch. Finally, the split pulses are recombined at the beam merger (BM). Note that we employed edge-polished crystals, instead of 10 µm thick Si(220) splitter crystals (Osaka *et al.*, 2013 ▸, 2016 ▸), to produce coherent split pulses with overlapped spectra. The shot-to-shot pulse energies of the split pulses are measured with transmissive beam intensity monitors (BIMs) placed in both delay branches. To vary the PLD between the two delay branches, the BRs are translated along the beam axes with motorized linear stages, as shown in Fig. 1 ▸(*a*). The maximum PLD is about 14 mm, which corresponds to a delay time of 47 ps at a photon energy of 10 keV, whereas it reaches 66 mm (220 ps) at 6.5 keV. More details of the interferometer with the wavefront splitters are found elsewhere (Hirano *et al.*, 2018 ▸).

The experiment was performed on BL3 at SACLA (Tono *et al.*, 2013 ▸) at 10 keV, for which the pulse duration has been estimated to be ∼8 fs full width at half-maximum (FWHM) from spectra measured with a wide-range high-resolution spectrometer (Inubushi *et al.*, 2017 ▸). The pulse energy of each delay branch was 0.18 µJ on average for an incident pulse energy of 7 µJ after the Si(111) double-crystal monochromator. The beam positions at the BM were adjusted by tuning the angles of the BS and BR2 with an imaging detector (BPM1) placed 0.4 m downstream from the BM. The two beams were horizontally separated at BPM1, while they were overlapped at the observation plane located 92 m downstream from the BM, where a high-resolution imaging detector (BPM2, 4 µm pixel^−1^) (Kameshima *et al.*, 2016 ▸) was placed. To observe interference fringes at BPM2, we precisely tuned the angles of the BM and controlled the angular deviation between the two beams. The delay time was coarsely adjusted to ± 500 fs using an X-ray streak camera (Hamamatsu, C4575-03) and was then finely tuned by observing interference fringes formed only at delay times less than the coherence time, as described below.

## 3. Results and discussion   

### 3.1. Visibility analysis   

**Figure 2:**
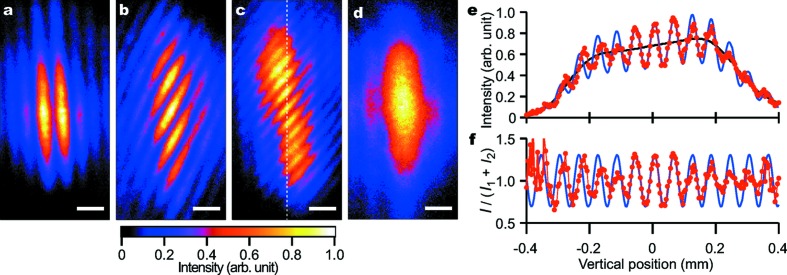
Examples of single-shot superimposed profiles, (*a*)–(*c*) with a near-zero delay and (*d*) with a delay far from zero. Each scale bar represents 100 µm. The angular deviations (α_*x*_, α_*z*_) for panels (*a*)–(*c*) evaluated with equation (1) are (2.0, 0.11), (1.7, −1.0) and (2.5, −2.1) µrad, respectively. The fringe profile shown in panel (*d*) originates from parasitic scattering from the edge of the BS and/or BM. (*e*) Line profile along the dashed line in panel (*c*) (symbols) and its low-pass filtered profile (black line). (*f*) Oscillatory component of the measured line profile (symbols) and a fitted cosine curve (blue line) with a fringe spacing δ_*z*_ of 60.0 µm and visibility *V* of 0.31. The fit is performed with a region in which the modulus becomes a maximum (near position 0 in this case). The fitted function multiplied by the low-pass filtered profile is also shown in panel (*e*) (blue line).

**Figure 3:**
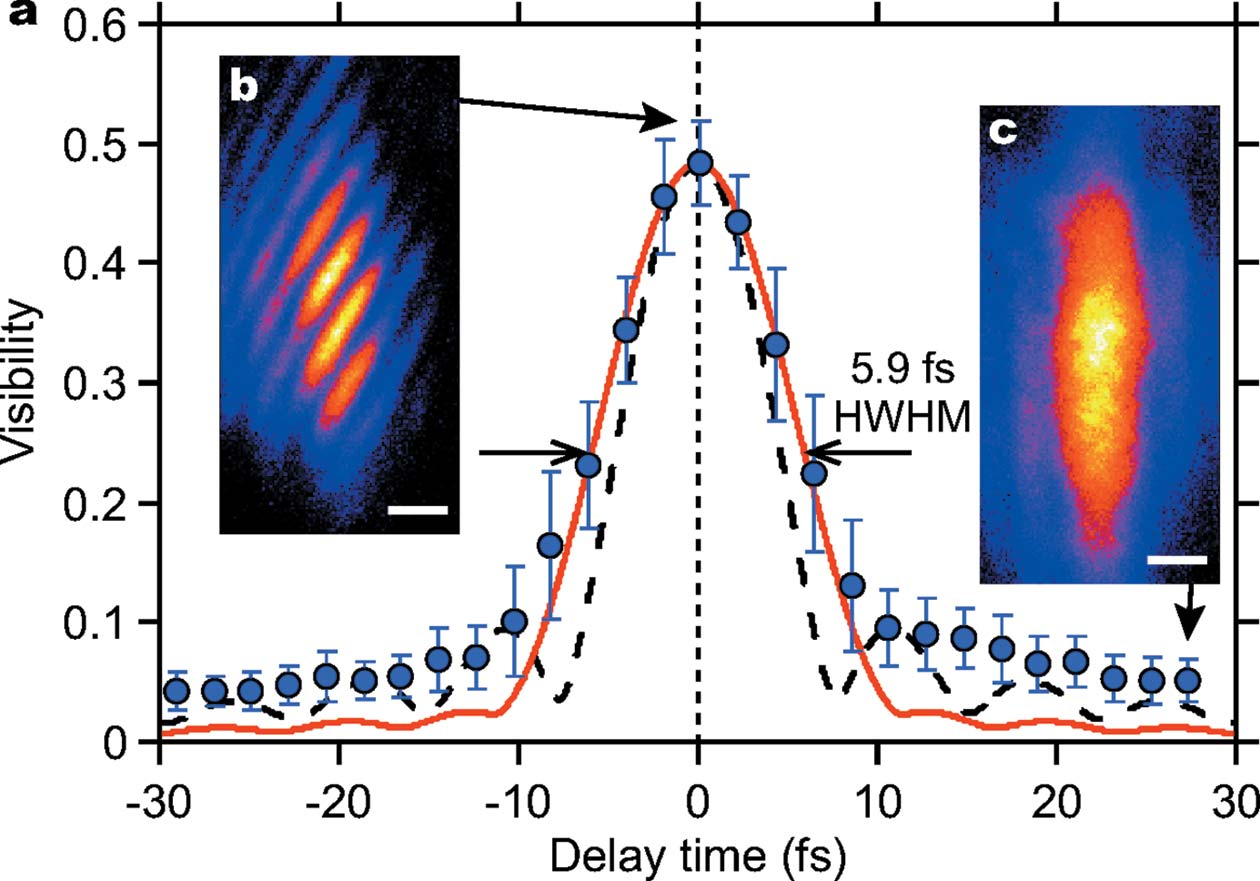
(*a*) Measured visibilities as a function of delay time. The average visibility at each delay time is displayed as filled circles. The error bars denote the standard deviations. The black dashed line indicates the modulus of the complex degree of coherence |γ_12_(τ)| calculated from the average spectrum of the exit beams composed of fourfold Si(220) diffractions. The red solid line represents the ensemble average of |γ_12_(τ)| calculated by considering Gaussian spectral spikes with a bandwidth of 375 meV (FWHM) and a fluctuation in the peak energy of 60 meV in the standard deviation. (*b*) Single-shot interference fringe with a maximum visibility of 0.55. (*c*) Superimposed profile measured at a delay far from zero for which the visibility is calculated to be 0.043. Each scale bar represents 100 µm.

Figs. 2 ▸(*a*)–2 ▸(*c*) show single-shot interferograms that were observed with a nearly zero delay. Under the assumption of the plane wave condition for the two beams, the interference fringe spacing in the horizontal (δ_*x*_) and vertical (δ_*z*_) directions can be written as
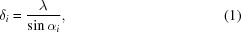
where *i* denotes the direction (*i* = *x*, *z*), α_*x*_ (α_*z*_) is the angular deviation between the two beams in the horizontal (vertical) direction and λ ≃ 1.24 Å is the wavelength. According to equation (1), vertical interference fringes were generated with only α_*x*_, as shown in Figs. 1 ▸(*a*) and 2 ▸(*a*), whereas an increase in α_*z*_ formed skew fringes (Figs. 2 ▸ *b* and 2 ▸ *c*), which allowed us to clearly distinguish interference fringes from unwanted parasitic fringes due to the scattering from the edges of the BS and BM (Fig. 2 ▸d), even for low visibility fringes. This measurement was repeated while changing the delay time τ. We found that the fringe visibility decreased for τ larger than 10 fs, as shown in Fig. 3.

To analyse the data quantitatively, the visibility of a single-shot interference fringe is calculated by the following procedure. An intensity profile along the vertical direction *I*(*z*) at the observation plane (BPM2) can be expressed as
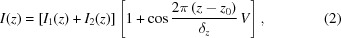
where *I*_1_(*z*) and *I*_2_(*z*) are the individual intensity profiles of the interfering beams, *z*_0_ the position with a phase difference between the beams of 2π*n* (*n* is an integer) and *V* the visibility. At sufficiently large τ with *V* = 0, the intensity profile *I*(*z*) is equal to the incoherent sum of the individual intensities, *I*_1_(*z*) + *I*_2_(*z*), while an oscillation of *I*(*z*) becomes pronounced at a larger *V*. The visibility *V* is associated with the complex degree of coherence γ_12_(τ) between wavefields *E*_1_ and *E*_2_ at a delay time τ, as follows (Goodman, 1985 ▸):
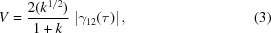
with
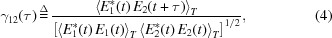
where *k* = *I*_1_(*z*)/*I*_2_(*z*) is the intensity ratio between the two beams at an observation point *z*, and the angle brackets 〈…〉*_T_* denote the time average over a time interval of *T*. For simplicity, we have here assumed that the incident beam has perfect transverse coherence. In this experiment, the effective time interval *T* is determined by the convolution of the pulse duration and the delay time, which is shorter than 100 ps. Equation (3) indicates that the visibility decreases while the intensity ratio *k* deviates from unity. If the intensity profile of the incident beam was spatially uniform, *k* would become unity over the whole superimposed area. However, we possibly had a spatial variation in *k* because we superimposed different portions of the incident beam with a lateral shift of approximately 100 µm under the present optical geometry [note that the original beam size was ∼500 µm (horizontal) × 400 µm (vertical) in FWHM]. Furthermore, the reduced longitudinal mode number with the SDO system could enhance fluctuations in the intensity, beam axis and profile of the output beams, which would lead to an increase in the variation of the splitting ratio. For simplicity in the analysis of visibility, we only utilized data sets with a splitting ratio ranging from 0.5 to 1.5 with reasonably high intensities for the two branches, where the profiles of the split beams were relatively similar to each other. The fraction of the data sets satisfying these requirements was approximately 10%. To evaluate the spatial variation in both *k* and *V* on a shot-by-shot basis, a low-pass filter was applied to each single-shot intensity profile, as shown in Fig. 2 ▸(*e*). Assuming that the incoherent sum *I*_1_(*z*) + *I*_2_(*z*) corresponds to the low-pass filtered profile, we extracted the oscillatory component from each intensity profile (Fig. 2 ▸ *f*), which clearly displays a spatial variation of modulus(*V*). Furthermore, we set the ratio *k* to unity at a region with the highest modulus. We consider that the latter assumption is valid because the averaged splitting ratio was ∼1. By fitting this region within equation (2), we obtained *V* for each single-shot fringe pattern for which *V* ≃ |γ_12_(τ)| should be a good approximation.

### 3.2. Evaluation of temporal coherence   

Fig. 3 ▸(*a*) displays the measured visibility as a function of delay time. We obtained a maximum visibility *V* of 0.55, as shown in Fig. 3 ▸(*b*). The deviation of *V* from unity can be explained partly by the transverse coherence. The two beams superimposed with a lateral shift of ∼100 µm in both directions denote a maximum visibility of ∼0.75 based on the experimental studies of transverse coherence previously performed at SACLA (Lehmkühler *et al.*, 2014 ▸; Inoue *et al.*, 2015 ▸), which indicate that the transverse coherence length is similar to the beam size. A further decrease in visibility may originate from incoherent parasitic scattering from the splitter edge, air, and dust on the polyimide films employed in the BIMs.

**Figure 4:**
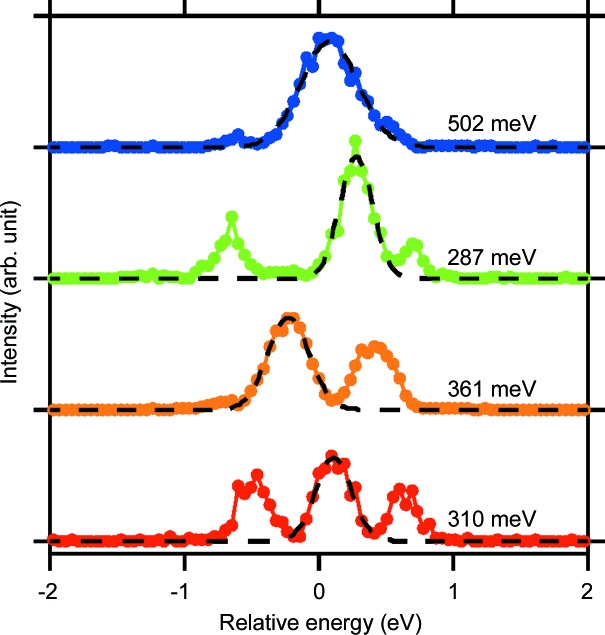
Typical single-shot spectra of incident XFEL pulses after the Si(111) double-crystal monochromator measured with a high-resolution dispersive spectrometer (Inubushi *et al.*, 2012 ▸; Katayama *et al.*, 2016 ▸) using an Si(660) flat crystal analyser. The energy resolution is approximately 50 meV. Each dashed line represents the fitted Gauss function, for which the FWHM is indicated. The number of longitudinal modes before the monochromator was approximately 60 (Inubushi *et al.*, 2017 ▸).

We evaluate the mean temporal coherence from the experimental results averaged over hundreds of shots at each delay time. The temporal width of the averaged visibilities, which corresponds to a coherence time τ_coh_, was determined to be τ_coh_ = 5.9 ± 0.7 fs in half-width at half-maximum. This τ_coh_ is close to the coherence time of 4.6 fs expected from the Fourier transform of the average spectrum of the exit beams calculated with fourfold Bragg-case Si(220) diffractions and is displayed as a black dashed line in Fig. 3 ▸(*a*). However, we observed a deviation of about 30% between the expected and measured coherence times. This can be attributed to the spiked structures in the XFEL spectra with a typical width of approximately 350 meV (FWHM) measured by a high-resolution dispersive spectrometer (Inubushi *et al.*, 2012 ▸; Katayama *et al.*, 2016 ▸), as shown in Fig. 4 ▸, which is narrower than the bandwidth of the Si(220) diffraction of 560 meV. In fact, an ensemble average of |γ_12_(τ)| calculated with a spike width of 375 meV (FWHM) shows a good agreement with the measured curve, as shown in Fig. 3 ▸(*a*). The spiked structures and resulting shot-to-shot spectral fluctuation may also increase the spread of the visibility values, especially at delay times near the mean coherence time. Therefore, the shot-by-shot spectral information, rather than the averaged information, should play an important role in this experimental scheme. Note that the tail part of the measured visibility curve could provide information on the pulse duration (Le Marec *et al.*, 2016 ▸). It is, however, difficult to distinguish true visibility values from artifacts in the analysis of low visibility fringes because considerably high visibility values of ∼0.05 were obtained through the analysis even far from the zero-delay condition, as shown in Fig. 3 ▸(*c*), due to unwanted high-frequency components in the profiles.

We have here characterized the mean temporal coherence of monochromatic XFEL pulses, which show similar properties to those expected from the bandwidth of the SDO system. Knowledge of the temporal coherence of original XFEL pulses is also important for a broad range of experiments with XFELs, although it requires interferometers with bandwidths Δ*E*/*E* of larger than 1%. Another type of hard X-ray interferometer employing multilayer mirrors with a broad bandwidth (Roling *et al.*, 2014 ▸) has the potential to characterize original XFEL pulses. The single-shot hard X-ray interferometry demonstrated in this study is applicable to various types of interferometers.

## 4. Future perspectives   

Finally, we discuss the future implications of the results obtained here. Although the maximum PLD was 9 µm in this study, this interferometer can produce a larger PLD of up to 14 mm at 10 keV. Future upgrades of the mechanics with the same optical configuration would enable the production of a PLD of 60 mm at 10 keV, while it would reach 120 mm at 5 keV. This interferometer can, therefore, be a powerful tool for characterizing the spectrum of highly monochromatic X-ray beams with an energy resolution below 10 µeV, which is beyond that obtained in state-of-the-art monochromators. Possible applications include ultra-high resolution X-ray spectroscopy, and characterization of high-resolution monochromators and narrow-band X-ray beams emitted from future sources such as XFEL oscillators (Kim *et al.*, 2008 ▸). Other applications involve the direct measurement of atomic scale dynamics at femtosecond to sub-nanosecond scales *via*, for example, X-ray photon correlation spectroscopy (XPCS) (Sutton *et al.*, 1991 ▸; Grübel *et al.*, 2007 ▸; Gutt *et al.*, 2009 ▸). The wide operational photon energy range from 6.5 to 11.5 keV allows the investigation of atomic fluctuation in diverse systems both at equilibrium and in transient (far from equilibrium) states.
